# Spatial Autocorrelation of Cancer Incidence in Saudi Arabia

**DOI:** 10.3390/ijerph10127207

**Published:** 2013-12-16

**Authors:** Khalid Al-Ahmadi, Ali Al-Zahrani

**Affiliations:** 1 King Abdulaziz City for Science and Technology, P.O. Box 6086, Riyadh 11442, Saudi Arabia; 2 King Faisal Specialist Hospital & Research Centre, P.O. Box 3354, Riyadh 11211, Saudi Arabia; E-Mail: alisaz@kfshrc.edu.sa

**Keywords:** cancer incidence, spatial autocorrelation, spatial pattern, spatial regression, geographical information system, Saudi Arabia

## Abstract

Little is known about the geographic distribution of common cancers in Saudi Arabia. We explored the spatial incidence patterns of common cancers in Saudi Arabia using spatial autocorrelation analyses, employing the global Moran’s *I* and Anselin’s local Moran’s *I* statistics to detect nonrandom incidence patterns. Global ordinary least squares (OLS) regression and local geographically-weighted regression (GWR) were applied to examine the spatial correlation of cancer incidences at the city level. Population-based records of cancers diagnosed between 1998 and 2004 were used. Male lung cancer and female breast cancer exhibited positive statistically significant global Moran’s *I* index values, indicating a tendency toward clustering. The Anselin’s local Moran’s *I* analyses revealed small significant clusters of lung cancer, prostate cancer and Hodgkin’s disease among males in the Eastern region and significant clusters of thyroid cancers in females in the Eastern and Riyadh regions. Additionally, both regression methods found significant associations among various cancers. For example, OLS and GWR revealed significant spatial associations among NHL, leukemia and Hodgkin’s disease (r² = 0.49–0.67 using OLS and r² = 0.52–0.68 using GWR) and between breast and prostate cancer (r² = 0.53 OLS and 0.57 GWR) in Saudi Arabian cities. These findings may help to generate etiologic hypotheses of cancer causation and identify spatial anomalies in cancer incidence in Saudi Arabia. Our findings should stimulate further research on the possible causes underlying these clusters and associations.

## 1. Introduction

Studies of the geographic variations in cancer mortality, prevalence and incidence have proven valuable for generating and evaluating etiologic hypotheses regarding cancer causation [1,2]. One method that has been especially useful in medical geographic research is spatial autocorrelation. Spatial autocorrelation can be defined as a situation in which the value of a variable at a specified geographic location depends on its values at adjacent locations [3].

Several studies have examined spatial autocorrelation, spatial patterns and associations at different cancer sites in different parts of the world using spatial statistical analysis techniques and regression models. La Vecchia and Decarli [4] examined the correlation patterns in the mortality rates attributed to 17 non-sexual and four sexual cancers in 20 Italian regions and found considerably higher rates of cancer at a number of common sites in northern areas. Rosenberg *et al*. [5] examined the distribution of mortality from 40 cancers in Western Europe using spatial autocorrelation techniques. They found that cancer mortality rates were strongly spatially correlated, implying a similar spatial arrangement of the responsible agents. They concluded that local spatial autocorrelation is a useful technique for exploring epidemiological maps. Another study investigated the relationships between pancreatic cancer incidence and 23 other cancer sites [6]. The findings of that study showed a highly significant association among the incidence rates of pancreatic, lung and kidney cancers for both genders; less consistent correlations were found for colorectal, endometrial, ovarian and bladder cancers. The researchers inferred that the association between pancreatic and lung cancer could be attributed to tobacco smoking and that the association with kidney cancer might reflect additional shared etiologic and pathogenetic risk factors for the two neoplasms. Mandal *et al*. [7] analyzed the correlation between female breast cancer and male prostate cancer in the United States between 2000 and 2005 using ordinary least squares regression (OLS) and geographically-weighted regression (GWR) analyses. Their findings suggested that breast and prostate cancers are spatially clustered, consistent with the results of other studies [8,9,10] that have identified comparable risk factors for these two cancers.

The literature suggests that some cancers share certain features and risk factors, and ecologic analyses of cancer incidence rates could support the formulation of hypotheses on these risk factors. Strong spatial association of cancer incidence rates might suggest that these variables are not spatially random, implying that the risk factors might be spatially associated. However, there have been no studies of the spatial pattern of cancer incidence in Saudi Arabia. This work attempts to fill this gap and lay the foundation for future studies of spatial cancer incidence. The findings of such analyses will be helpful to recognize cancer patterns, leading to hypotheses on the etiology and mechanisms of cancers.

The purpose of this study was twofold: (i) to explore the spatial patterns and clusters of the most common cancers in Saudi Arabia using global and local spatial autocorrelation analyses (*i.e*., by calculating the global Moran’s *I* statistic and the Anselin local Moran’s *I* statistic) and (ii) to examine whether the incidence rates of the most common cancers are spatially correlated at the city level using bivariate OLS regression and GWR. Since performing multiple comparisons (*i.e*., pairwise tests) on a single set of data increases the risk of obtaining false-positive results (Type I errors), the significance levels in this research will be adjusted by applying the Bonferroni correction (BC).

## 2. Materials and Methods

### 2.1. Cancer, Population and Spatial Data

This study covers all cancer cases diagnosed among Saudi citizens between January 1998 and December 2004 that were recorded in the Saudi Cancer Registry (SCR). The data included in this study were retrieved in May 2008; therefore, cases that were identified after this date were not used in this project. However, we anticipate that the number of cases reported later will be proportionately related to the number of sources that report cases and the efficiency of case reporting in each region. Cancer data were received from the SCR, which is a population-based registry whose primary goal is to define the population-based incidence of cancer in Saudi Arabia. Cancer has been made a mandatory notifiable disease by the Saudi Ministry of Health to ensure comprehensive data collection. The SCR has full access to cancer data from all public and private hospitals, as well as clinics and laboratories throughout the country. Cancer data are abstracted by trained cancer registrars from patients’ medical records, laboratory and histopathology reports, clinical notes, radiology reports, and death notifications and death certificates: therefore, case findings are based on several data sources, which enhanced case ascertainment and ensured the completeness of data to provide reliable national cancer statistics. This study focused only on Saudi nationals. Cancer management is offered free of charge to all Saudi patients, including those who may need further treatment abroad, regardless of their socioeconomic status or place of residency. It is therefore unlikely for a Saudi citizen to seek treatment outside the national healthcare system. In this research, we are studying the correlation between cancer occurrence (incidence) and place of residency. Although major cancer treatment centers are located in the major regions (Riyadh, Makkah, and Eastern regions), cancer detection and diagnosis is usually made at the secondary healthcare centers, which are widely spread to cover all geographic regions of the country. While inequitable access to an advanced healthcare system for cancer patients from remote areas is expected, we believe that this would have negative impacts on the treatment outcome rather than on cancer detection, given that we have based our analyses on population-based cancer data for the period between 1998 and 2004, which is subject to continuous updates and regular adjustments. Thus, we believe that the data reflect the actual distribution of cancer cases in all regions.

The crude incidence rate (CIR) was calculated using standard methods [11]. The age-standardized incidence rate (ASR) takes into account the age structures of the populations in geographical zones or areas to provide unbiased comparisons of cancer incidence. Population data by age group category were not available at the city level; however, such data were available at the region level. Thus, only the crude rate for cities was computed in the present study. A spatial database of cancer incidence in Saudi Arabia was designed and developed in the form of an ESRI Geodatabase. To develop the database, individual cancer cases were aggregated at the city level. A detailed description of the cancer data, population data, spatial data, mapping methods and cancer rates used in this study can be found in Al-Ahmadi *et al*. [12].

### 2.2. Spatial Patterns and Cluster Analyses

The distribution of any phenomenon (e.g., cancer) or its associated values (e.g., incidence rate) within a space will produce a pattern. The geographic patterns range from completely clustered at one extreme to completely dispersed at the other. Patterns that fall between these extremes are assumed to be random. Knowing whether there is a pattern is useful for gaining a better understanding of a geographic phenomenon, monitoring conditions on the ground, comparing patterns or tracking changes [13]. The first “law” of geography, which states that “everything is related to everything else, but near things are more related than distant things” [14], is a crucial idea in geography and particularly in spatial data analysis. In statistical terms, this law is related to the concept of spatial autocorrelation [15]. Positive spatial autocorrelation indicates that neighboring values are similar, suggesting spatial dependency; negative spatial autocorrelation indicates that neighboring values are dissimilar, suggesting inverse spatial dependence. An autocorrelation value of zero implies that there is no spatial pattern. According to Kalkhan [16], the most commonly used measures of spatial autocorrelation in ecological, health, environmental and geological studies are Moran’s *I* statistic [17], Geary’s *C* statistic [18] and the spatial cross-correlation statistic [19]. These measures use the sizes of feature values (such as the incidence rates of cancer in cities) to detect and quantify the significance of spatial patterns. However, Moran’s *I* and Geary’s *C* are global statistics (*i.e*., they estimate the overall degree of spatial autocorrelation). In this study, both the global Moran’s *I* statistic and the Anselin local Moran’s *I* statistic were applied to explore the spatial patterns of the most common cancers in Saudi Arabia.

Global Moran’s *I* measures the spatial autocorrelation of feature locations (*i.e*., cities in this study) and feature attributes or values (*i.e*., cancer incidence rate) simultaneously. Given a set of features and an associated attribute, the measure evaluates whether the pattern is clustered, dispersed, or random [20]. To explore the overall spatial patterns of the most common cancers in Saudi Arabia, the global Moran’s *I* statistic was used to represent the degree of clustering. It is calculated as follows [21]:

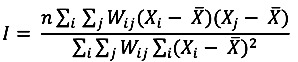
(1)
where:

*X_i_* = the crude incidence rate of cancer for the *i*th city;
*X* the mean crude incidence rate of cancer for all of the cities in the study area;
X*_j_* = the crude incidence rate of cancer for the *j*th city;
*W_ij_* = a weight parameter for the pair of cities *i* and *j* that represents proximity; and
*n* = the number of cities.

Thus, *I* ˃ 0 indicates a clustered pattern (*i.e*., similar values are found together), *I* = 0 indicates a random pattern, and *I* < 0 indicates a dispersed pattern (*i.e*., high and low values are scattered). The Anselin local Moran’s *I* is a local spatial autocorrelation statistic based on Moran’s *I* statistic. This statistic was developed by Anselin [22] as a local indicator of spatial association (LISA). According to Anselin, LISA statistics have the following two properties: (i) the LISA for each observation suggests the extent of significant spatial clustering of similar values around that observation, and (ii) the sum of the LISAs for all observations is proportional to the global spatial association. The Anselin local Moran’s *I* was calculated for each city in the study area to explore spatial clusters of similar crude incidence rates. The Anselin local Moran’s *I* (*Ii*) for the *i*th city is calculated according to Anselin [22]:

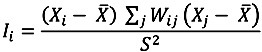
(2)
where:

*X_i_* = the crude incidence rate of cancer for the *i*th city;
*X* = the mean crude incidence rate of cancer for the cities in the study area;
X*_j_* = the crude incidence rate for the *j*th city;
*W_ij_* = a weight parameter for the pair of cities *i* and *j* that represents proximity; and
*S* = the standard deviation of the crude incidence rate of cancer in the study area.

The Anselin local Moran’s *I* identifies statistically significant (at a 95% confidence level; *p* < 0.05) spatial clusters of cities with high or low crude cancer incidence rates. Clusters of cities with high crude cancer incidence rates (high-high or HH) are considered “hotspots”, whereas clusters of cities with low crude cancer incidence rates (low-low or LL) are considered cold spots. In addition, the Anselin local Moran’s *I* identifies cities with high crude cancer incidence rates that are surrounded mainly by cities with low crude cancer incidence rates (high-low or HL) and cities with low crude cancer incidence rates that are surrounded chiefly by cities with high crude cancer incidence rates (low-high or LH). The cut-off distance at which the overall degree of clustering was maximized was used to calculate the Anselin local Moran’s *I*.

Since performing multiple comparisons (*i.e*., pairwise tests) on a single set of data increases the risk of obtaining false-positive results (Type I errors), in this research the significance levels will be adjusted by applying the Bonferroni correction (BC). The BC is an adjustment made to *p* values when several dependent or independent statistical tests are being performed simultaneously on a single data set. To perform a BC, we divide the critical *p* value (α) by the number of comparisons being made. For example, if ten hypotheses are being tested, the new critical *P* value would be α/10. The statistical power of the study is then calculated based on this modified *p* value [23].

### 2.3. Modeling Spatial Relationships

Beyond investigating how geographic features are distributed and clustered, spatial analysis can be used to examine the relationships between features. In the context of this work, measuring and identifying the relationships between cancer incidence rates at the city level are of particular interest. Global relationships between two or more variables are commonly explored using techniques such as ordinary least squares (OLS) regression, in which the relationship can be expressed as an equation that defines the best fit for the line. To examine the relationship between two cancer incidence rates, one cancer is considered the dependent variable, *y*, and another is considered the independent variable, *x*:

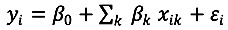
(3)

GWR is a relatively simple technique that extends the traditional regression framework of Equation (3) by allowing the estimation of local rather than global parameters; in GWR, the model is rewritten as follows:

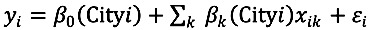
(4)
where the dependent variable *y* is regressed on a set of independent variables, each denoted by *x_k_*, and the parameters are allowed to vary in space. Herein, (*u_i_*, *v_i_*) denotes the longitude and latitude coordinates of the *i*th city, and *β_k_*(City*i*) is a representation of the continuous surface of parameter values. Measurements of this surface are taken at a set of points to determine the spatial variability of the surface [24]. As explained earlier, in this research the significance levels will be adjusted by applying the Bonferroni correction.

## 3. Results

This study explored the spatial patterns and clusters of the most common cancers in Saudi Arabia using global and local spatial autocorrelation analyses. It also examined whether the incidence rates of the most common cancers are spatially correlated at the city level using bivariate OLS regression and GWR. Table 1 shows the number of diagnosed cases of the most common cancers in Saudi Arabia. SCR registered a total of 45,532 cancer cases diagnosed among Saudi nationals during the period between January 1998 and December 2004. From those, a total of 22,930 (50.3%) were males and 22,602 (49.7%) were females. Liver cancer was the commonest tumor, accounting for 8.84% of the total cancers in males, followed closely by Non-Hodgkin’s lymphoma (NHL) with 8.80% and leukemia with 8.19%. Colorectal cancer ranked fourth, followed by lung and prostate cancer. In females, breast cancer was the commonest cancer, accounting for 20.2% of total cancers, followed by thyroid cancer with 9.3%. Colorectal cancer ranked third, closely followed by NHL and leukemia.

**Table 1:** Number of diagnosed cases of the most common cancers.

Cancer Site	All	Male	Female
Breast	4,668	106	4,562
NHL	3,483	2,018	1,465
Colorectal	3,322	1,763	1,559
Leukemia	3,286	1,879	1,407
Liver	2,831	2,027	804
Thyroid	2,695	595	2,100
Lung	1,867	1,464	403
Other skin	1,660	957	703
Hodgkin’s Disease	1,616	982	634
Bladder	1,425	1,122	303
Prostate	1,290	1,290	0
Ovary	786	0	786
Cervix uteri	641	0	641

Given that 111 cities in Saudi Arabia were included in this research, spatial autocorrelations were computed for ten different distances or distance classes, beginning at 50 km and increasing in increments of 50 km. Thus, the minimum distance was 50 km and the maximum distance was 500 km. The z-score was computed at each distance to determine the intensity of spatial clustering. The statistically significant z-scores indicated the scales at which the spatial clustering was most pronounced. One approach for detecting an appropriate scale of analysis is to select the distance associated with the statistically significant peak that best reflects the scale of the analysis. This peak is commonly the first statistically significant peak. The peaks reflect the distances at which the spatial processes that promote clustering are most pronounced.

**Figure 1:**
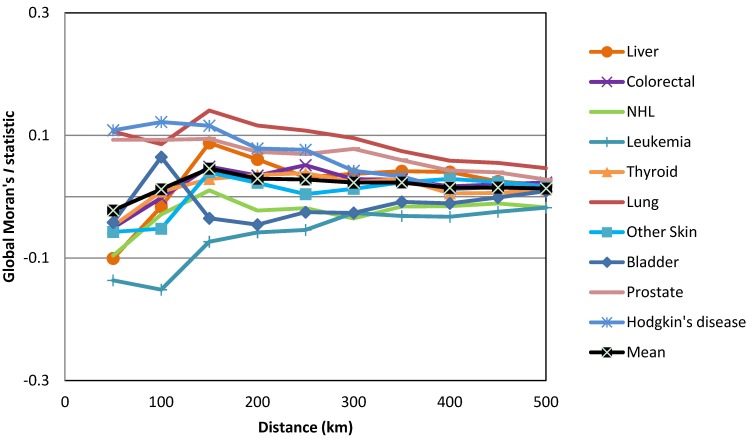
Global Moran’s *I* statistics for the most common cancers in males.

**Figure 2:**
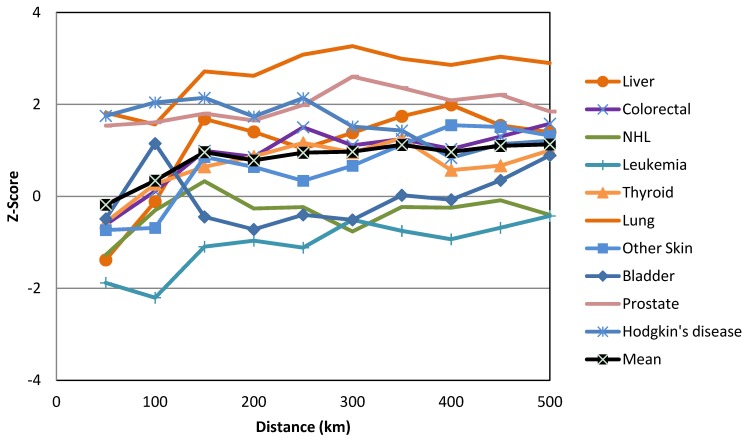
Z-scores of the global Moran’s *I* statistics for the most common cancers in males.

**Figure 3:**
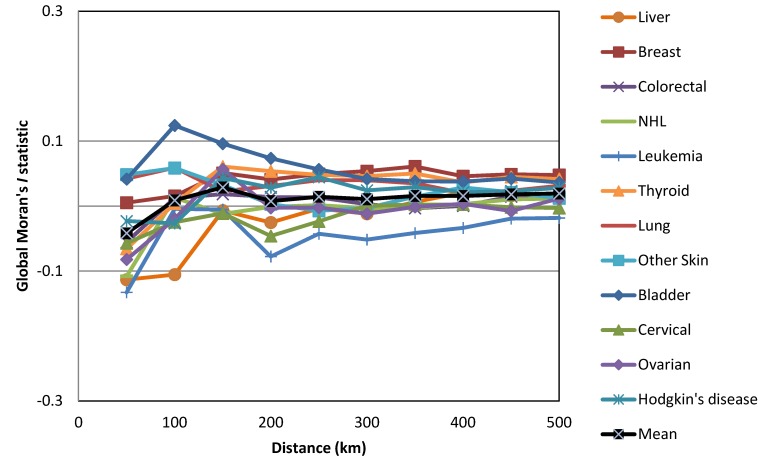
Global Moran’s *I* statistics for the most common cancers in females.

**Figure 4:**
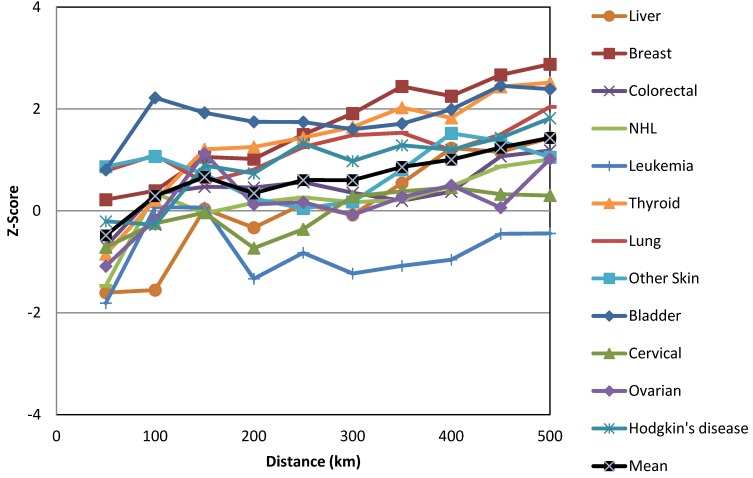
Z-scores of the global Moran’s *I* statistics for the most common cancers in females.

The only global Moran’s *I* statistic values that we considered in this study were those that were statistically significant according to the Bonferroni correction at a confidence level of 95% (−1.96 ˃ z-score > +1.96, where *p* < 0.05). As shown in Figure 1, the global Moran’s *I* statistics for the most common cancers among males varied across neighborhood distances (cut-off distances) ranging from 50 km to 500 km. However, only lung cancer was statistically significant according to the Bonferroni correction at *p* < 0.005. Lung cancer in males produced the most positive statistically significant global Moran’s *I* statistic, which was clustered at a range of neighborhood sizes (250 km to 500 km) (Figure 2). Among females, only breast cancer was statistically significant according to the Bonferroni correction at *p* < 0.004 at the neighborhood distance of 500 km (Figure 3). Breast cancer among females exhibited the greatest positive statistically significant global Moran’s *I* statistics, indicating a clustered pattern (Figure 4).

**Figure 5:**
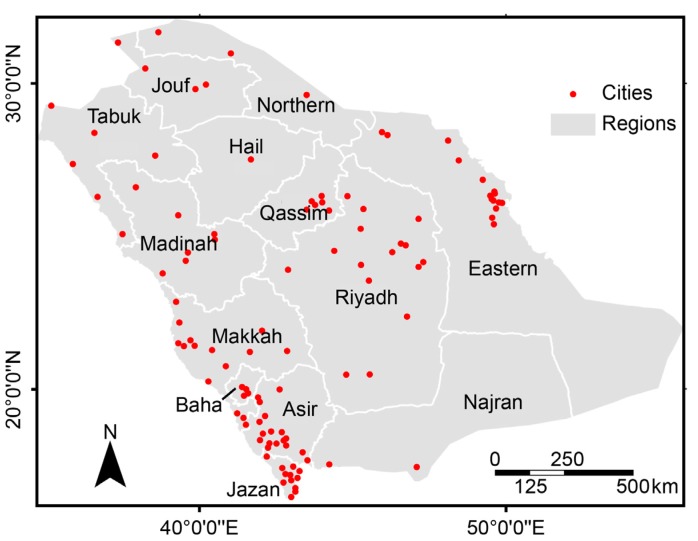
Regions and cities of Saudi Arabia.

Local statistics identify spatial variation in the relationships between variables. These statistics are particularly useful for identifying geographic clusters (*i.e*., hotspots), for testing assumptions of heterogeneity and for determining the distance beyond which the spatial relationships between variables cease [22,25]. Figure 5 shows the locations of the cities included in this study and the Saudi administrative regions. Table 2 summarizes the Anselin local Moran’s *I* (ALMI) statistics for the most common cancers for each gender that were registered between 1998 and 2004 among Saudi citizens in Saudi Arabia. The ALMI identifies statistically significant (at a 95% confidence level, *p* < 0.05) spatial clusters of cities with high or low crude incidence rates (CIRs). A statistically significant positive ALMI value indicates that the surrounding cities have similar CIRs: *i.e*., a city with a high CIR in an area with a high CIR (high-high or HH) or a city with a low CIR in an area with a low CIR (low-low or LL). ALMI also highlights outliers: a statistically significant negative ALMI value indicates that a city has a different CIR from its neighbors: *i.e*., a city with a high CIR that is surrounded chiefly by cities with low CIRs (high-low or HL) or a city with a low CIR that is surrounded chiefly by cities with high CIRs (low-high or LH).

Overall, the results suggest that there is no statistically significant spatial autocorrelation of the most common cancers in Saudi Arabia; 86.5 to 97.3% of the cities exhibited no statistically significant spatial autocorrelation. The highest statistically significant positive HH ALMI value was found for bladder and thyroid cancers in females; 8.1% of the cities exhibited spatial clusters of high incidence rates for both of these cancers. The spatial clusters of female thyroid cancer were in the Riyadh and the Eastern regions; the clusters of bladder cancer were in the Jizan and Asir regions in the southern part of Saudi Arabia.

Among males, lung cancer, prostate cancer and Hodgkin’s disease exhibited the highest statistically significant positive HH ALMI values; 6.3% of the cities in the Eastern region had spatial clusters of high incidence rates of these three cancers. Hodgkin’s disease was clustered in the Qassim region. Approximately 4.5% of the cities in the Eastern and Qassim regions exhibited spatial clusters of male thyroid cancer. Female breast and cervical cancers were clustered in the Eastern region; male colorectal cancer was clustered in the Eastern, Qassim and Riyadh regions; male liver cancer was clustered in the Riyadh and Qassim regions; and female Hodgkin’s disease was clustered in the Eastern and Riyadh regions. The other common cancers were associated with statistically significant positive HH ALMI values ranging between 0.9 and 3.6% in the Riyadh, Eastern, Qassim, Jizan and Asir regions.

**Figure 6:**
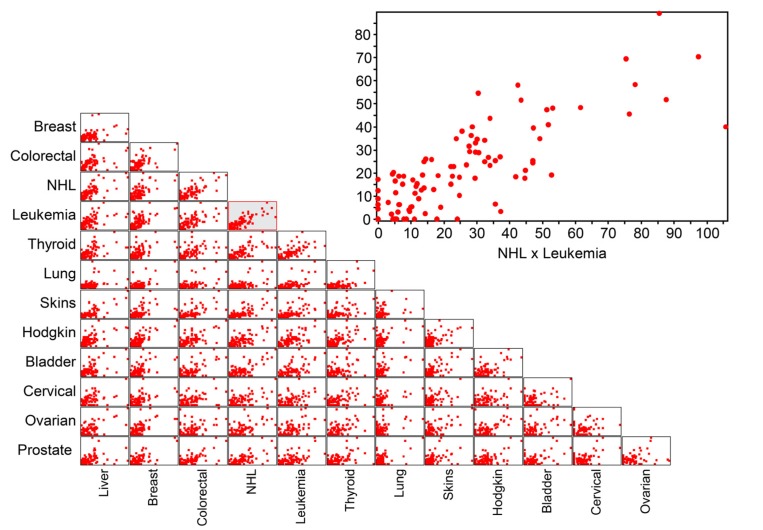
Scatterplot matrix of the incidence rates of the most common cancers, highlighting NHL and leukemia (inset).

Figure 6 shows a scatterplot matrix that displays the relationships between the incidence rates of the most common cancers in Saudi Arabia’s cities. Overall, there is a spatial relationship among the most common cancers. However, OLS and GWR were applied to explore the spatial relationships among the most common types of cancer diagnosed in Saudi Arabia between 1998 and 2004. Whereas the global OLS provided a global regression model for the whole study area (*i.e*., Saudi Arabia), the GWR produced a local regression equation for each city.

**Table 2:** Anselin local Moran’s *I* (ALMI) statistic results.

Cancers	Gender	HH		HL		LH		LL		Not Sig.		Regions with HH Clusters
		No.	%	No.	%	No.	%	No.	%	No.	%	
Liver	Male	5	4.5	3	2.7	3	2.7	0	0	100	90.1	Riyadh, Qassim
	Female	1	0.9	2	1.8	0	0	0	0	108	97.3	Riyadh
Colorectal	Male	5	4.5	2	1.8	1	0.9	0	0	103	92.8	Eastern, Qassim, Riyadh
	Female	1	0.9	3	2.7	0	0	0	0	107	96.4	Qassim
NHL	Male	1	0.9	5	4.5	1	0.9	0	0	104	93.7	Riyadh
	Female	1	0.9	2	1.8	0	0	0	0	108	97.3	Eastern
Leukemia	Male	2	1.8	3	2.7	0	0	0	0	106	95.5	Qassim
	Female	0	0	1	0.9	0	0	0	0	110	99.1	None
Thyroid	Male	6	5.4	4	3.6	0	0	0	0	101	91	Eastern, Qassim
	Female	9	8.1	4	3.6	2	1.8	0	0	96	86.5	Riyadh, Eastern
Lung	Male	7	6.3	1	0.9	3	2.7	0	0	100	90.1	Eastern
	Female	4	3.6	3	2.7	0	0	0	0	104	93.7	Eastern
Other skin	Male	4	3.6	0	0	0	0	0	0	107	96.4	Asir, Jizan, Qassim
	Female	2	1.8	3	2.7	0	0	0	0	106	95.5	Jizan
Bladder	Male	2	1.8	2	1.8	0	0	0	0	107	96.4	Asir
	Female	9	8.1	0	0	0	0	0	0	102	91.9	Jizan, Asir
Hodgkin’s disease	Male	7	6.3	4	3.6	1	0.9	4	3.6	95	85.6	Eastern, Qassim
	Female	6	5.4	3	2.7	2	1.8	0	0	100	90.1	Eastern, Riyadh
Breast	Female	5	4.5	3	2.7	4	3.6	0	0	99	89.2	Eastern
Cervical	Female	5	4.5	5	4.5	4	3.6	0	0	97	87.4	Eastern
Ovarian	Female	4	3.6	6	5.4	4	3.6	0	0	97	87.4	Riyadh
Prostate	Male	7	6.3	2	1.8	3	2.7	0	0	99	89.2	Eastern

In terms of the coefficient of determination, our results showed that the OLS regression revealed statistically significant spatial associations among the most common cancers in Saudi Arabia (Table 3). The significance levels were adjusted by applying the Bonferroni correction. The strongest association was between NHL and leukemia (r² = 0.67), and the weakest association was between liver and cervical cancers (r² = 0.24). Eight of the thirteen most common cancers in Saudi Arabia were highly associated with NHL: the r² values were 0.67 for leukemia, 0.65 for colorectal cancer, 0.59 for other skin cancers, 0.58 for both bladder and liver cancers, 0.55 for thyroid cancer, 0.53 for Hodgkin’s disease and 0.51 for breast cancer. Almost 50% of the most common cancer sites were associated with breast cancer, with r² values of 0.61 for Hodgkin’s disease, 0.53 for prostate cancer, 0.52 for colorectal cancer, 0.51 for leukemia and 0.50 for both ovarian and lung cancers. In contrast, the weakest associations were found between cervical cancer and other types of cancer: the lowest r² was 0.24 for liver cancer, and the highest r² was 0.41 for prostate cancer.

In contrast, more pronounced associations among the most common cancers were found in the GWR analyses compared with the OLS results (Table 4). The strongest association was between lung cancer and cervical cancer (r² = 0.88), and the lowest association was between cervical cancer and ovarian cancer (r² = 0.20). Breast cancer and Hodgkin’s disease exhibited strong associations with the other seven most common cancer types, with r² values ranging from 0.61 to 0.75. Breast cancer was strongly associated with other skin cancers (r² = 0.75), ovarian cancer (0.74), Hodgkin’s disease (0.65), colorectal and bladder cancers (0.63), NHL (0.58) and leukemia (0.51). Hodgkin’s disease had strong associations with other skin cancers (r² = 0.70), liver cancer (0.70), ovarian cancer (0.69), breast cancer (0.65), bladder cancer (0.63), NHL (0.61) and leukemia (0.52). In contrast, weak associations were found between cervical cancer and the other most common cancer types: the lowest was for ovarian cancer (r² = 0.09) and the highest was for prostate cancer (r² = 0.41).

**Table 3:** Coefficients of determination (r²) derived from OLS models.

Cancers	Liver	Breast	Colorectal	NHL	Leukemia	Thyroid	Lung	Other Skin	Bladder	Cervical	Ovarian	Prostate	Hodgkin’s Disease
Liver	1												
Breast	0.28 *	1											
Colorectal	0.35 *	0.52 *	1										
NHL	0.58 *	0.51 *	0.65 *	1									
Leukemia	0.41 *	0.48 *	0.51 *	0.67 *	1								
Thyroid	0.39 *	0.37 *	0.35 *	0.55 *	0.48 *	1							
Lung	0.16	0.50 *	0.32 *	0.29 *	0.34 *	0.31 *	1						
Other skin	0.63 *	0.34 *	0.49 *	0.59 *	0.41 *	0.32 *	0.16	1					
Bladder	0.35 *	0.41 *	0.48 *	0.58 *	0.43 *	0.25 *	0.29 *	0.54 *	1				
Cervical	0.24 *	0.31 *	0.30 *	0.36 *	0.26 *	0.29 *	0.18	0.19	0.20	1			
Ovarian	0.49 *	0.50 *	0.30 *	0.46 *	0.43 *	0.37 *	0.28 *	0.42 *	0.37 *	0.09	1		
Prostate	0.19	0.53 *	0.46 *	0.48 *	0.40 *	0.30 *	0.35 *	0.19	0.31 *	0.41 *	0.20	1	
Hodgkin’s disease	0.43 *	0.61 *	0.50 *	0.53 *	0.49 *	0.45 *	0.38 *	0.40 *	0.43 *	0.31 *	0.42 *	0.37 *	1

* Statistical significance (*p* < 0.05) adjusted by applying the Bonferroni correction.

**Table 4:** Coefficients of determination (r²) derived from GWR models.

Cancers	Liver	Breast	Colorectal	NHL	Leukemia	Thyroid	Lung	Other Skin	Bladder	Cervical	Ovarian	Prostate	Hodgkin’s Disease
Liver	1												
Breast	0.54	1											
Colorectal	0.50	0.63	1										
NHL	0.74	0.58	0.66	1									
Leukemia	0.42	0.51	0.52	0.68	1								
Thyroid	0.55	0.39	0.37	0.57	0.49	1							
Lung	0.52	0.56	0.34	0.46	0.61	0.47	1						
Other skin	0.70	0.75	0.77	0.72	0.58	0.57	0.74	1					
Bladder	0.57	0.63	0.60	0.61	0.46	0.34	0.71	0.64	1				
Cervical	0.37	0.43	0.34	0.39	0.28	0.32	0.88	0.31	0.34	1			
Ovarian	0.50	0.74	0.47	0.58	0.49	0.53	0.73	0.63	0.59	0.20	1		
Prostate	0.50	0.57	0.49	0.54	0.44	0.33	0.58	0.33	0.42	0.44	0.39	1	
Hodgkin’s disease	0.70	0.65	0.57	0.61	0.52	0.46	0.56	0.70	0.63	0.39	0.69	0.48	1

**Figure 7:**
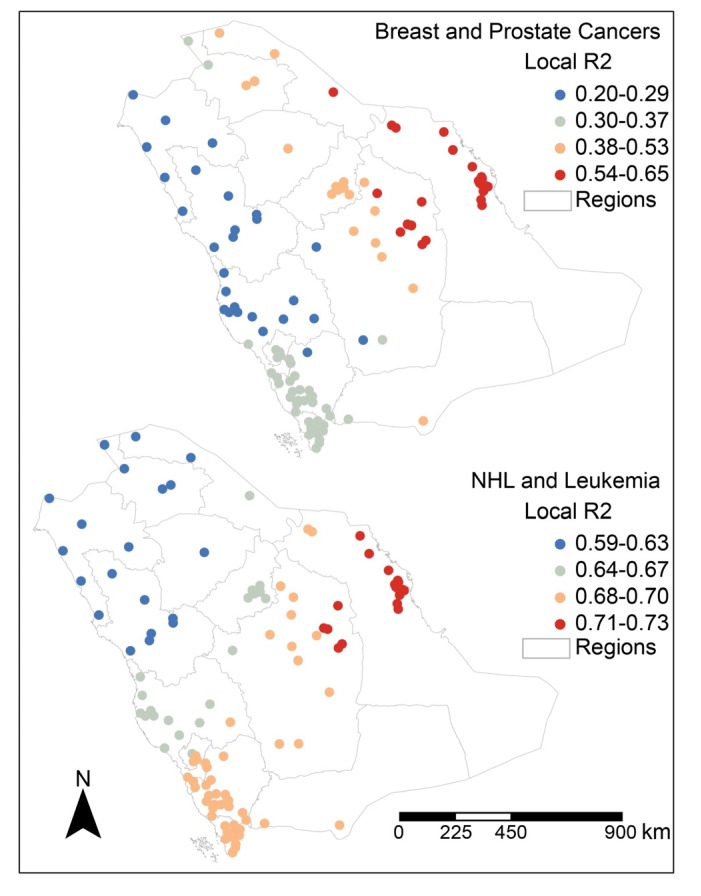
Spatial distribution of local coefficients of determination (r²) derived from GWR models.

Figure 7 shows the distribution of the local coefficient r² derived from the GWR models for the spatial association between NHL and leukemia and between breast and prostate cancers. Cities in the Eastern region and some cities in the Riyadh region exhibited the highest local correlation coefficients between breast and prostate cancers (r² = 0.54–0.65), and low associations were found in the northwestern, mid-western and southwestern regions of the country. High local correlation coefficients for the relationship between NHL and leukemia were also observed in most cities in the Eastern region and in some cities in the Riyadh region. However, the central and southwestern regions produced moderate associations, and the lowest associations were found in the northern regions.

## 4. Discussion

In an effort to explore and explain the spatial patterns of the most common cancers in Saudi Arabia, spatial autocorrelation techniques were applied to empirical cancer data. Two specific techniques, one global and one local, were applied at the city level. The former technique used the global Moran’s *I* statistic, while the latter used the Anselin local Moran’s *I* statistic. The global techniques yielded one metric that provided a summary of the cancer pattern over the entire study area, whereas the local techniques identified the spatial variations in cancer incidence between cities and were particularly useful for identifying cancer clusters or hot spots. The findings from the global and local spatial autocorrelation techniques used in this study revealed similarities and differences among the regions.

Among males, the global spatial autocorrelation analysis found a cluster of lung cancer only, while the local spatial autocorrelation analysis found clusters of lung cancer, prostate cancer and Hodgkin’s disease. The local spatial autocorrelation analysis suggested that these three cancers were clustered in cities in Eastern region. Lung cancer was the leading cause of death from cancer among Saudi males [26] and was ranked the fifth most common cancer during this study period (1998–2004). In 2002, lung cancer was the third leading cause of death in men and the tenth leading cause of death in women in Saudi Arabia [27]. Tobacco smoking is the single most important risk factor for cancer, especially lung cancer [28,29]. Since 1970, the prevalence of smoking has increased in Saudi Arabia, and this probably led to a lung cancer epidemic [30]. A recent prospective study of lung cancer in Saudi Arabia found that 71.1% of the patients were smokers; 95.1% of these were male; and the duration of smoking and the number of packs per year were the two chief risk factors related to the incidence of lung cancer [31]. Eastern region has the highest proportion (35.7%) of smokers among all Saudi regions [32]. Thus, the clustering of lung cancer in Eastern region can partly be attributed to the high prevalence of smoking. Air pollution may also increase the risk of lung cancer, and this risk is further increased for smokers [33,34,35]. In Eastern region, a strong association was observed between the concentration of NO_2_ air pollution and the risk of developing lung cancer [36]. Although age is the main risk factor for prostate cancer among males, the increased incidence of prostate cancer is probably a result of increased prostate-specific antigen (PSA) screening [37]. Prostate cancer was the sixth most common type of cancer in males in Saudi Arabia. The cluster found in the Eastern region can partly be explained by differences in the availability of PSA screening. A PSA screening program for employees and their dependents was established by Saudi ARAMCO (one of the largest oil company in the world, which has branches in most of the major cities in the Eastern region) in 1995, while the Ministry of Health hospitals in Saudi Arabia have much lower rates of PSA testing [38,39]. The reason for a possible Hodgkin’s disease cluster in Eastern region is unclear. Further investigation is required to determine why clusters of lung cancer, prostate cancer and Hodgkin’s disease appear and why these clusters are present in Eastern region.

Among females, the global spatial autocorrelation analysis found a cluster of breast cancer only, whereas the local spatial autocorrelation analysis found that the most significant clusters were for thyroid and bladder cancers. Thyroid cancer was the second most common cancer among females in Saudi Arabia, after breast cancer. The local spatial autocorrelation analysis found that female thyroid cancer was clustered in Riyadh and Eastern regions. Epidemiological studies have revealed that increased iodine consumption, irradiation, increased parity and regular high seafood consumption may be related to the etiology of thyroid cancer [40,41,42,43,44,45]. A study in a Middle Eastern population with relatively high birth and fertility rates and a high incidence of thyroid cancer supported the hypothesis that reproductive factors, mainly childbearing at older ages and high parity, may contribute to the risk of developing thyroid cancer [46].

To achieve the second aim of this research, OLS and GWR techniques were applied to explore the spatial relationships between the most common types of cancer diagnosed in Saudi Arabia between 1998 and 2004. Whereas the global OLS provided a global regression model for the whole study area (*i.e*., Saudi Arabia), the GWR produced a local regression equation for each city. Both the OLS and GWR regression findings identified significant spatial associations among cancers in Saudi Arabia; however, the GWR results revealed more pronounced associations than did the OLS results. This finding implies that the GWR models explained more of the spatial variation in the associations among cancer incidence rates in cities than did the OLS models. It is possible that GWR achieved better results than did the global OLS in this study because GWR distinguished spatial variations in the relationships among types of cancer, whereas the OLS model masked these variations. Moreover, because the observed spatial distributions of cancer incidence were non-stationary (*i.e*., they varied from one city to another), the global OLS model would probably not have detected a large proportion of the local variation, which might explain the poor results of the global OLS model.

The literature states that some cancers share certain features, characteristics and risk factors. Furthermore, the strong spatial association of the cancer incidence rates clearly indicates that these variables are not spatially random. This result would imply that the risk factors might be spatially associated, but it does not directly identify these factors. These factors could be endogenous or exogenous, and determining which of these risk factors might be significant requires further research. An increase in cell division induced by exogenous or endogenous factors is the root of the pathogenesis of all human cancers [47]. Although the causes of most types of cancer are only partially understood, at least a proportion of cases can be explained by endogenous factors (such as genetic, behavioral and cultural risk factors; ethnic or regional cultural differences in foods, drinks or sexual practices) or exogenous factors (such as industrial pollution; the intensity and duration of sunlight; the use of hormone therapies or contraceptives; the consumption of dietary fats; and environmental endocrine disruptors). The findings in the current work imply that cancers that are strongly spatially associated might share similar risk factors.

According to the OLS and GWR results, there was a significant spatial association between female breast cancer and male prostate cancer (r² = 0.53 and 0.57, respectively) in Saudi Arabian cities. This result could be partially explained by genetic risk factors. López-otín and Diamandis [9] compared female breast cancer with male prostate cancer and highlighted several similar features and characteristics; one similarity is that both are hormonally regulated. Some of the key genetic mutations associated with breast cancer, such as BRCA1 and BRCA2 (human genes that belong to a class of genes known as tumor suppressors), have also been found in some individuals with prostate cancer [8]. Moreover, Mandal *et al*. [7] suggested that female breast cancer and male prostate cancer were spatially correlated at the county level in the United States. Epidemiological studies have also found another relationship between these two cancers: relatively high rates of breast cancer were found in the relatives of early-onset prostate cancer patients [10]. There were also significant spatial associations between NHL, leukemia and Hodgkin’s disease (r² = 0.49–0.67 using OLS and r² = 0.52–0.68 using GWR). These three cancers are hematologic cancers (*i.e*., cancers of the blood and bone marrow), and this association might indicate shared risk factors. Most cities in the Eastern region exhibited the highest coefficient of determination for these three cancers. The Eastern region includes Saudi ARAMCO, one of the largest oil company in the world, and Jubail Industrial City, a global hub for chemical industries and one of the largest industrial city in the Middle East. Branches of these two companies are distributed throughout the major cities of the Eastern region. Empirical studies have highlighted that exposure to petroleum emissions and petrochemicals and proximity to the petroleum oil industry are associated with leukemia, NHL and lung cancer [48,49,50,51,52,53]; however, further investigation is needed to determine whether this association also occurs in Saudi Arabia. One study has shown an increased risk of liver cancer in people with a father and brother diagnosed with prostate cancer. Our results demonstrated a spatial association between liver cancer and prostate cancer (r² = 0.50 using GWR). However, further genetic, environmental and socioeconomic investigations could address the gap in knowledge about the causes of these cancers and could improve our understanding about their epidemiology.

One limitation of the present study is that it is an ecological study, in which cancer incidence cases were analyzed as geographic units rather than individual cases. Conducting the spatial statistical analysis with individual cancer cases and finer-level geographic units would offer more detailed information because analyses of the relationship between health and place can be affected by the scale and zoning design used [54]. Another issue associated with the aggregated cancer incidence rates is the method by which the geographic boundaries of the statistical areas are defined; this difficulty is known as the modifiable areal unit problem (MAUP) [55] and has been noted previously in health-related studies [54,56,57,58].

## 5. Conclusions

At present, little is known about the spatial pattern of the most common cancers in Saudi Arabia. This study is the first to explore the spatial incidence patterns of the most common cancers in Saudi Arabia using spatial autocorrelation analyses. Furthermore, it is the first to examine whether the incidence of the most common cancers is spatially correlated at the city level using both global and local regression methods. Overall, the results suggest that there is no statistically significant spatial autocorrelation among the most common cancers in Saudi Arabia; the majority of the cities exhibited no statistically significant spatial autocorrelation. However, the global Moran’s *I* statistics showed that male lung cancer and female breast cancer exhibited positive statistically significant associations according to the Bonferroni correction, indicating a clustered pattern. Anselin’s local Moran’s *I* analyses revealed small significant clusters of lung cancer, prostate cancer and Hodgkin’s disease among males in the Eastern region and significant clusters of thyroid cancers in females in the Eastern and Riyadh regions. Both the OLS and GWR regression findings showed that there were significant spatial associations among cancers in Saudi Arabia; however, the GWR results revealed more pronounced associations than did the OLS results. There are few previous ecological studies related to spatial cancer incidence rates and their relationships to various risk factors in Saudi Arabia. We believe that this study of the spatial clustering and spatial associations between the most common cancers in Saudi Arabia could prompt further studies of the spatial epidemiology of cancer. The clusters and associations of the most common cancer types identified in this study could be used to generate etiologic hypotheses of cancer causation, suggesting obvious etiologies, providing support for or against present hypotheses, suggesting places and scales for upcoming epidemiological research, identifying spatial anomalies in the epidemiology of cancers, identifying hot spots and revealing spatiotemporal patterns in cancer incidence. Our results should stimulate further ecologic and etiological research on the possible causes underlying the clusters and spatial associations of the most common cancers in Saudi Arabia.
